# The Role of Oxidative Stress Enhanced by Adiposity in Cardiometabolic Diseases

**DOI:** 10.3390/ijms24076382

**Published:** 2023-03-28

**Authors:** Iwona Świątkiewicz, Marcin Wróblewski, Jarosław Nuszkiewicz, Paweł Sutkowy, Joanna Wróblewska, Alina Woźniak

**Affiliations:** 1 Department of Cardiology and Internal Medicine, Collegium Medicum, Nicolaus Copernicus University, 85-094 Bydgoszcz, Poland; 2 Division of Cardiovascular Medicine, University of California San Diego, La Jolla, CA 92037, USA; 3 Department of Medical Biology and Biochemistry, Collegium Medicum, Nicolaus Copernicus University, 85-092 Bydgoszcz, Poland

**Keywords:** oxidative stress, obesity, cardiovascular disease, cardiometabolic diseases, coronary artery disease, metabolic syndrome, type 2 diabetes

## Abstract

Cardiometabolic diseases (CMDs), including cardiovascular disease (CVD), metabolic syndrome (MetS), and type 2 diabetes (T2D), are associated with increased morbidity and mortality. The growing prevalence of CVD is mostly attributed to the aging population and common occurrence of risk factors, such as high systolic blood pressure, elevated plasma glucose, and increased body mass index, which led to a global epidemic of obesity, MetS, and T2D. Oxidant–antioxidant balance disorders largely contribute to the pathogenesis and outcomes of CMDs, such as systemic essential hypertension, coronary artery disease, stroke, and MetS. Enhanced and disturbed generation of reactive oxygen species in excess adipose tissue during obesity may lead to increased oxidative stress. Understanding the interplay between adiposity, oxidative stress, and cardiometabolic risks can have translational impacts, leading to the identification of novel effective strategies for reducing the CMDs burden. The present review article is based on extant results from basic and clinical studies and specifically addresses the various aspects associated with oxidant–antioxidant balance disorders in the course of CMDs in subjects with excess adipose tissue accumulation. We aim at giving a comprehensive overview of existing knowledge, knowledge gaps, and future perspectives for further basic and clinical research. We provide insights into both the mechanisms and clinical implications of effects related to the interplay between adiposity and oxidative stress for treating and preventing CMDs. Future basic research and clinical trials are needed to further examine the mechanisms of adiposity-enhanced oxidative stress in CMDs and the efficacy of antioxidant therapies for reducing risk and improving outcome of patients with CMDs.

## 1. Introduction

Cardiometabolic diseases (CMDs), such as cardiovascular disease (CVD), metabolic syndrome (MetS), and type 2 diabetes (T2D), are associated with increased morbidity and mortality [1,2]. CVD, including coronary artery disease (CAD) and systemic essential hypertension (HTN), are among the main causes of premature and excess mortality in developed countries [1]. CAD is a leading single cause of death in people over 50 years of age [1]. HTN remains a major cardiovascular risk factor, almost doubling the risk of death, with a rising systolic and diastolic blood pressure (BP) of as much as 20 and 10 mmHg, respectively [3,4]. Moreover, while the overall prevalence of HTN in the adult population is ~30–45% globally, HTN becomes progressively more common with advancing age, reaching >60% in people aged >60 years [3]. MetS, which occurs in approximately 25–30% of adults, doubles the long-term risk of developing CVD and is associated with a 5-fold increase in the risk of T2D [2,5]. The incidence of T2D is constantly growing, with an increase in deaths from T2D by 70% globally between 2000 and 2019 [1]. The increasing prevalence of CMDs has been attributed to the aging population and the common occurrence of risk factors, such as high systolic BP, elevated plasma glucose, and increased body mass index (BMI) [1]. The global epidemic of obesity, MetS, and T2D in adult and children populations developed over the last decades [1]. Notably, elevated fasting plasma glucose and high BMI are among the leading risk factors that displayed the largest increases in risk exposure over the period from 1990 to 2019 [6].

The CMDs are characterized by coexistence of multiple risk factors, including excess weight and adiposity, dyslipidemia, insulin resistance, high fasting plasma glucose, impaired glucose tolerance or T2D, cigarette smoking, elevated BP, physical inactivity, and erratic dietary patterns, all of which contribute to the pathogenesis of CMDs and impact patient outcomes [2,7,8,9,10]. The leading risk factor globally for attributable deaths is high systolic BP, which accounts for ~10.8 million deaths (i.e., ~19% of all deaths) and has emerged as the most important risk factor in older people [6]. Specifically, high systolic BP accounts for 9.3% of the disability-adjusted life years (DALYs) in the entire population and 6.0%, 16.1%, and 19.5% for subgroups with ages 25–49 years, 50–74 years, and ≥75 years, respectively. The marked rise of prevalence of high fasting plasma glucose and high BMI, and their large contribution to CMDs burden, is particularly alarming, which is reinforced by insufficient understanding of underlying mechanisms. For example, the prevalence of high BMI is rising significantly faster than prevalence of low physical activity, excessive caloric intake, and poor diet quality, all of which contribute to high BMI [6]. Identifying and addressing risk factors can reduce cardiometabolic risks; however, the efficacy of currently used preventive and therapeutic strategies is insufficient [8].

Obesity is associated with an increased risk of metabolic disorders, including MetS and T2D, and is among the major risk factors for CVD [1,2,9,11]. Obesity-related mortality and disability are caused mainly by CVD [1]. The prevalence of obesity increased during the past three decades at a faster pace than the related disease burden [1,12]. In the adult US population, the prevalence of overweight and obese was reported as high as 71% and 40%, respectively [2]. Overweight or obesity occurs in ~83%, ~76%, and ~74% of subjects with T2D, HTN, and dyslipidemia, respectively [13]. Importantly, ~33% of overweight and ~65% of obese individuals fulfilled the criteria for MetS [14]. The prevalence of abdominal obesity manifesting as an elevated waist circumference, which is a typical feature of MetS, was reported in the adult US population at ~56% [15]. Additionally, an increased waist circumference was the most common abnormality in 34,821 subjects with MetS, mostly from the European countries enrolled in the Metabolic syndrome and Arteries Research (MARE) Consortium [9].

Understanding the underlying mechanisms and identifying risk factors for CMDs plays a significant role in reducing cardiometabolic risk and improving patient outcome. Oxidative stress, which results from a lack of balance between oxygen derivatives generation and their removal by the antioxidant defense system, contributes to the pathophysiology of obesity, atherosclerosis, and CMDs [10,16,17,18,19]. The significance of oxidative stress relates to the fundamental role of reactive oxygen species (ROS) and redox signaling in molecular, cellular, and systems processes [20,21]. In obesity, enhanced and disturbed generation of reactive oxygen species (ROS) in excess adipose tissue (AT) may lead to increased oxidative stress. Oxidant–antioxidant imbalance is a common feature of various CMDs, including HTN, CAD, stroke, and MetS. Oxidative stress can result in various disorders, such as endothelial damage, vascular dysfunction, cardiovascular remodeling, and systemic inflammation [17,19]. In addition, oxidative stress is shown to be associated with impaired insulin signaling pathways and insulin resistance [17,18,19,22].

The mechanisms and clinical implications related to the interplay between adiposity, oxidative stress, and CMDs have not been comprehensively addressed [23,24,25,26,27]. The present review article is based on extant results from basic and clinical studies, and specifically addresses the various aspects associated with oxidant–antioxidant balance disorders in the course of CMDs in subjects with excessive accumulation of AT and obesity. We aim to give a comprehensive overview of existing knowledge about associations between adiposity-enhanced oxidative stress and cardiometabolic risks to indicate knowledge gaps and offer future perspectives for further basic and clinical research. We provide insights into both the mechanisms and clinical implications of effects related to the interplay between adiposity and oxidative stress for treating and preventing CMDs.

## 2. Characteristics of Adipose Tissue

Adipose tissue (AT) is one of the main types of loose connective tissue [28]. Adipocytes constitute the main fraction of AT-building cells [29]. In addition to adipocytes, there are also stromal vascular fraction, adipose-derived stem cells, preadipocytes, macrophages, lymphocytes, eosinophils, mast cells, fibroblasts, and nerve cells [27,30,31]. There are four types of AT differentiated by histological structure and function: white adipose tissue (WAT), brown adipose tissue (BAT), beige adipose tissue, and pink adipose tissue [27,32]. WAT, one of the largest organs, is the main energy store of the organism, which captures and accumulates lipids [33]. By collecting triacylglycerols and glucose, WAT protects other tissues [33]. Adipocytes that make up WAT are characterized by a significant lipid content [34]. High lipid content in WAT acts as a thermal insulator and helps maintain internal body temperature [35]. WAT also produces and releases a variety of bioactive molecules, including the adipokines, which are biologically active proteins with a low molecular weight synthetized and secreted mainly by WAT [36]. These biomolecules exhibit autocrine, paracrine, and endocrine effects on tissues [37]. So far, over 600 adipokines were detected and described in scientific literature [38]. The main role of adipokines is to regulate metabolism and bioenergetic homeostasis [39]. Moreover, adipokines have immunomodulatory properties [40]. In the course of obesity, a change in the adipokine profile is observed in favor of the increased secretion of pro-inflammatory adipokines with a simultaneous reduction in the level of anti-inflammatory adipokines [41,42]. This leads to chronic low-grade inflammation, which affects not only AT, but also other tissues [43]. BAT is made up of adipocytes containing many fat droplets of varying sizes [44]. Compared to WAT, BAT is characterized by a large number of mitochondria in adipocytes [45]. A significant number of mitochondria enables the implementation of the main BAT function: non-shivering thermogenesis [46]. Beige AT is a transition form between WAT and BAT [47]. It is formed as a result of the beiging of WAT adipocytes [48]. Beige adipocytes acquire the properties that are typical for BAT, and their role is also changed, from cells constituting an energy store to energy-releasing adipocytes [49]. The main factor leading to the formation of beige AT is chronic exposure to low temperatures [46]. Pink AT is formed in mammary gland alveolar epithelial cells [50,51]. This tissue is involved in the production and secretion of milk during lactation [52].

AT is characterized by high plasticity and adaptation to changing conditions [46]. Not only are the type and volume of AT important, but also the location. In clinical terms, visceral AT is extremely significant. The increase in visceral AT volume results in abdominal obesity and an increased risk of CMDs [53]. Visceral AT is formed mainly by WAT and is a source of adipokines [54]. Epicardial AT, which is a particular form of visceral AT, participates in the pathogenesis of CAD, atrial fibrillation (AF), and heart failure (HF) with preserved left ventricular (LV) ejection fraction [55,56]. Additionally, the association between perivascular AT (PVAT) and the occurrence of CVD was found [57]. In the course of obesity, PVAT hypertrophy and hyperplasia are observed [58]. PVAT expansion leads to atheromatous plaque development and vascular calcification [59]. Under physiological conditions, a positive effect of PVAT on cardiovascular homeostasis is observed in patients with an AT amount within normal limits [60,61]. Subcutaneous AT is the second largest depot of fat in the human body [62]. The amount of subcutaneous AT is proportional to visceral AT and increases in the course of obesity [63]. Recent studies indicated that subcutaneous AT participates in the regulation of lipid-carbohydrate metabolism [64].

Obesity is defined as a state of excessive accumulation of AT that exceeds the adaptive abilities of the organism and increases the risk of developing other diseases [65,66]. Obesity is the global epidemic affecting more than 2.3 billion people worldwide, both adults and children [1,9].

In clinical practice, the calculation of BMI is the most common method of diagnosing excess weight, including obesity [67]. A BMI value of 18.5–24.9 kg/m^2^ was determined as a normal value, while values of 25.0–29.9 kg/m^2^ and ≥30.0 kg/m^2^ indicate overweight and obesity, respectively [68]. Patients with BMI values of 30.0–34.9 kg/m^2^ are diagnosed with obesity class I, BMI of 35.0–39.9 kg/m^2^ is considered as obesity class II, and BMI ≥ 40 kg/m^2^ is defined as obesity class III [68]. However, population- and country-specific criteria should be considered. For example, the optimal cut-off point for the identification of metabolic disorders in the Polish population is 27.2 kg/m^2^ [69]. Additionally, for an equivalent age-adjusted and sex-adjusted obesity-based risk of T2D at a BMI of 30.0 kg/m^2^ in White populations, the lower BMI cutoffs for South Asian (23.9 kg/m^2^), Black (28.1 kg/m^2^), Chinese (26.9 kg/m^2^), and Arab (26.6 kg/m^2^) populations were found [70].

Increased waist circumference, which indicates the presence of excess central (abdominal) obesity, is a typical finding in subjects with MetS and is common in patients with other CMDs [5,71]. For the increased waist circumference, the population-, ethnic-, gender-, and country-specific definitions should be used. The cut-off values of increased waist circumference for different populations are provided in Table 1 [5,71]. The waist circumference measurement is recommended for those with a BMI of 25 to 34.9 kg/m^2^ to provide additional information on CVD risk; however, if BMI is >30 kg/m^2^, central obesity can be assumed and waist circumference does not need to be measured [5,71]. Nevertheless, owing to the need for screening of individuals with a metabolically obese normal weight, the measuring of waist circumference should be considered when BMI is ≥22.5 kg/m^2^ in females and ≥23.8 kg/m^2^ in males [14,69,72].

Waist–hip ratio (WHR), another indicator of abdominal obesity, is calculated as the ratio of the waist circumference to the hip circumference [73]. The WHR reference values are gender specific. For males, physiologically WHR is >0.90 and for females >0.85 [74].

BMI, waist circumference, and WHR are often used in clinical practice due to the simplicity of measurement and calculations, while other methods, such as bioelectrical impedance, are used to determine the content of AT in the body [75]. It is estimated that in healthy adult males and healthy adult females, fat should account for 17.6–25.3% and 28.8–35.7% of body mass, respectively [76].

Obesity is closely related to the MetS that is associated with adverse outcome [2,5,9,71,77,78]. MetS is defined as a set of interrelated factors that significantly increase the risk of other CMDs, including T2D and CVD [5,9,10,71,78,79,80,81]. Abdominal obesity is among the diagnostic criteria for MetS [80]. According to the International Diabetes Federation (IDF) criteria, MetS is defined in the presence of ≥3 of the following five risk factors: increased waist circumference (population- and country-specific definitions should be used), elevated fasting plasma glucose (≥100 mg/dL or drug treatment for this disorder), elevated BP (systolic ≥130 and/or diastolic ≥85 mmHg or antihypertensive drug treatment), hypertriglyceridemia (≥150 mg/dL or drug treatment for this disorder), and reduced high-density lipoprotein cholesterol (HDL-C) (<40 mg/dL in males and <50 mg/dL in females or drug treatment for this disorder) [5,71]. Most T2D patients met the MetS criteria [5,71]. MetS occurs in ~25% of adults depending on age, gender, race, country of origin, and diagnostic criteria [2,5,9,14,71,82,83,84]. For example, in the National Health and Nutrition Examination Survey (NHANES) [14,82,83], MetS was diagnosed in ~34% of US adults 20 years of age and over. Importantly, while MetS occurred in 16% of females and 20% of males under 40 years of age, 52% of males and 54% of females 60 years of age and over met the MetS criteria [14]. The pathogenesis of MetS is influenced by genetic and lifestyle factors [85]. In patients with MetS, lifestyle modifications, and in some cases, pharmacological treatment, are required for improving patient outcome [3,74]. A lack of effective intervention in patients with MetS may lead to developing other CMDs, such as CVD, disability, and premature death [84].

## 3. The Oxidant–Antioxidant Balance and Oxidative Stress

Reactive oxygen species (ROS) and reactive nitrogen species (RNS) are the products of normal cellular metabolism [20,21]. The main source of ROS in the cell is a mitochondrial respiratory chain. ROS are also generated in peroxisomes, endoplasmic reticulum, and during reactions catalyzed by xanthine oxidase, endothelial oxidases, and phagocyte-reduced nicotinamide adenine dinucleotide phosphate (NADPH) oxidase (NOX) [86,87,88]. Nitric oxide (^.^NO) is, in turn, generated from L-Arginine with the participation of nitric oxide synthases (NOS) [89]. ROS/RNS in the living system play a double role. They are not only deleterious species, but also act as “second messengers” by participating in a number of normal regulatory processes [89,90,91]. It was shown that redox signaling may control cellular functions through modifications of the activity of enzymes participating in metabolic processes, regulation of gene expression, transcription factors, and through the impact on the nature of the epigenetic modifications [92]. The key redox signaling agents are hydrogen peroxide (H_2_O_2_) and the superoxide anion radical (O_2_^−^) [93]. O_2_^−^ is considered a primary ROS, participating in both signaling and in cell injury [94]. As a result of the reactions with the participation of this radical, other ROS are generated, such as H_2_O_2_ and hydroxyl radical (^.^OH) [95]. The manner of ROS/RNS activity depends mainly on their concentration [86]. Occurring in low/moderate concentrations, ROS show beneficial effects [20]. At high concentrations, however, they may damage all major cellular components [96]. Specifically, they participate in the oxidation of proteins, carbohydrates, lipids, and DNA, causing damage to DNA, cellular membranes, and organelles [97].

Under physiological conditions, intracellular ROS homeostasis is subject to strict control, resulting in exceptionally low levels of ROS in the cell [90]. Complex mechanisms, with which aerobic organisms are equipped, protect against excessive ROS generation [98,99,100,101]. In order to maintain redox balance, their activity encompasses prevention, interception, and repair [102]. It was proven that the main role in antioxidant protection is played by enzymes [103], such as superoxide dismutase (SOD), glutathione peroxidase (GPx), catalase (CAT), glutathione reductase (GR), and xanthine oxidoreductase (XOR) [104]. Redox homeostasis of the cell is also maintained by non-enzymatic ROS/RNS scavengers, including both endogenous antioxidants, such as glutathione, ferritin, ceruloplasmin, uric acid, and coenzyme Q and exogenous antioxidants, such as carotenoids, polyphenolic compounds, vitamin C, and vitamin E [105,106,107].

Lack of balance between ROS/RNS generation and their removal by the antioxidant defense system leads to oxidative stress [16]. It can result from excessive generation of these oxygen and/or nitrogen derivatives or from weakening activity of antioxidant mechanisms [105]. More recent publications suggest two ways of classifying oxidative stress: time based and concentration/intensity based [90]. Based on time criterion, we can distinguish “acute” and “chronic” oxidative stress [90]. The classification based on its intensity indicates basal, low intensity, intermediate intensity, and high intensity oxidative stress [108]. One of the most significant consequences of oxidative stress is enhancement of lipid peroxidation, which results in the oxidation of polyunsaturated fatty acids (PUFAs), which are part of cellular membranes [109]. The products of this process are conjugated dienes (CD) and lipid peroxides (ROOH) [109], and also the so-called secondary products of lipid peroxidation, such as malondialdehyde (MDA), 4-hydroxy-2-nonenal (4-HNE) [110], and isoprostanes (IsoPs) [111]. It was shown that oxidative stress may take part in the etiopathogenesis of numerous systemic diseases, including CMDs such as CVD [16,86,97] and MetS [112,113].

## 4. Adipose Tissue as a Source of Free Radicals

The main endogenous sources of ROS in AT are mitochondria and NOX. Key importance is also given to NOS, Fenton’s reaction, microsomal cytochrome P450, peroxisomal β-oxidation, as well as lipoxygenases and cyclooxygenases [114].

The results of in vitro studies show that during the basic state of mitochondria activity, 0.2–2% of oxygen is converted in a respiratory chain into ROS [115], mainly from complex I and complex III (originally O_2_^−^) [116]. Complex I receives electrons from reduced nicotinamide adenine dinucleotide (NADH), which leads to a premature leak of electrons and penetration of ROS to the mitochondrial matrix. In complex III, however, ROS are generated on both sides of the inner mitochondrial membrane [117]. In obesity, mitochondria are particularly susceptible to ROS generation because when an excess of nutrients occurs in adipocytes and mitochondrial substrates, the ROS concentration increases [118]. In a cell culture of 3T3-L1 adipocytes, it was shown that high concentrations of glucose or free fatty acids increase ROS generation in mitochondria [119]. It is considered that the excess of ROS in mitochondria leads to their dysfunction, which is the cause of T2D, non-alcoholic fatty liver disease, HF, and MetS. Other studies also suggest a possibility of developing myocarditis caused by changes in immunological response due to mitochondrial damage to DNA by free radicals [120].

NOX is a membranous protein that transports electrons from NADPH to O_2_, the side effect of which is ROS generation in the cytoplasm. Among seven NOX isoforms identified in mammalian adipocytes, the most numerous isoform is NOX4 [114], although its exceptionally strong expression was also noted in the cardiovascular system (together with NOX1 and NOX5 isoforms) [121]. In cell cultures, it was shown that NOX4 expression and the resulting ROS generation increased in adipocytes exposed to excess glucose or palmitic acid salts [122,123]. NOX4 is also distinguished by the fact that it generates H_2_O_2_, which penetrates through membranes and is a more durable form of ROS in comparison with O_2_^−^, produced by the other NOX isoforms. What is also significant for AT is NOX2 isoform, because it dominates in the cellular membrane of macrophages of AT and generates O_2_^−^ in response to lipopolysaccharides or saturated fatty acids [124].

In obese individuals, ROS sources in AT can change from NOX4 at an early stage to NOX2 at a medium stage, and then passing to the late stage mainly into impaired oxidative phosphorylation in mitochondria [125]. With reference to vascular stroma cells, macrophages seem to be of greatest significance for ROS sources in AT. They are even considered to be the main factor regulating activity of AT in free radical signaling pathways [124].

**Figure 1:**
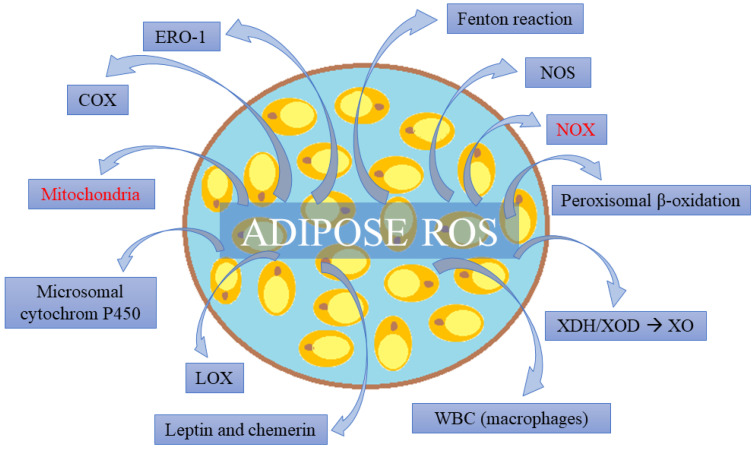
Adipose tissue as a source of reactive oxygen species (ROS). The main sources are mitochondria and reduced nicotinamide adenine dinucleotide phosphate (NADPH) oxidase (NOX). (ERO-1) endoplasmic reticular oxidoreductin 1, (COX) cyclooxygenase, (LOX) lipoxygenase, (WBC) white blood cells, (XDH) xanthine dehydrogenase, (XOR) xanthine oxidoreductase, (XO) xanthine oxidase, and (NOS) nitric oxide synthase.

A significant endogenous ROS source in adipocytes can also be endoplasmic reticular oxidoreductin 1 (ERO1) and the xanthine dehydrogenase (XDH)/oxidoreductase (XOD) system (Figure 1). ERO1 is a protein disulfide oxidase of endoplasmic reticulum, which generates H_2_O_2_ as a result of protein folding and secretion [124]. The XDH/XOD system becomes an ROS source under oxidative stress conditions, which is, for example, observed during obesity. XOD then changes into xanthine oxidase (XO) and generates O_2_^−^ and H_2_O_2_ in a series of catabolic reactions of purine conversion into uric acid [23].

Fisher-Wellman and Neufer [116], moreover, propounded a hypothesis that under insulin resistance conditions, when a decreased concentration of glutathione (GSH) is observed, pyruvate dehydrogenase and nicotinamide nucleotide transhydrogenase can be a significant source of H_2_O_2_ in adipocytes. Enhanced oxidative redox signaling in AT, as it was already mentioned mainly in obese individuals, results in further peroxidative consequences for the organism, being a result of this tissue disorder [124]. Apart from the abovementioned mechanisms, it is mainly about abnormal adipokine secretion into the bloodstream. In the obese state, leptin [126] and chemerin [127] are secreted in excess (see Figure 1), whereas adiponectin [128] and omentin-1 [129] are subject to decreased secretive activity of AT. This leads to oxidative stress and an inflammatory state in vascular endothelium, and by the same token, dysfunction of blood vessels (HTN, atherosclerosis-typical for MetS). In the context of the present paper, what is of particular significance is PVAT; since it is adjacent to blood vessels, it affects them directly. PVAT in obese individuals has a vasoconstrictive activity, whereas in a healthy organism, it relaxes the smooth muscle of blood vessels [23].

## 5. Oxidative Stress in Cardiometabolic Disorders in Subjects with Obesity

**Figure 2:**
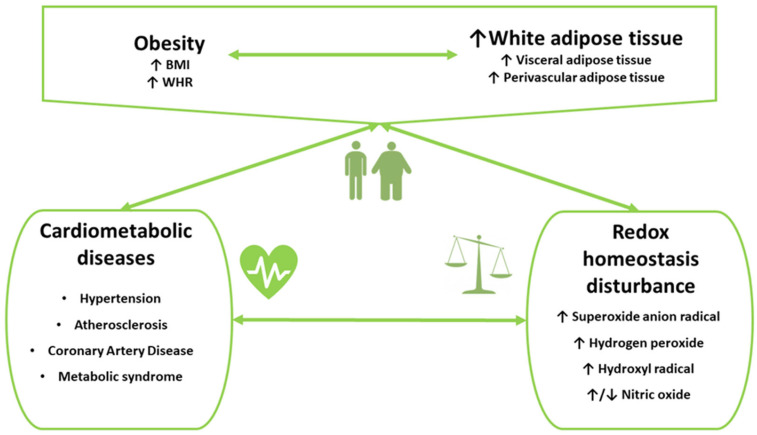
Demonstrated and potential associations between adiposity, redox homeostasis disorders, and cardiometabolic diseases.

Oxidative stress is a common feature of CMDs including various types of CVD, such as HTN and atherosclerotic CVD, and MetS (Figure 2). The interplay between obesity, oxidative stress, and low-grade chronic inflammation in the course of CMDs is linked to adverse outcomes, such as premature and accelerated process of significant atherosclerosis, CAD events including acute myocardial infarction, post-infarct LV dysfunction and remodeling, and HF in long-term follow-up [130,131,132,133,134,135,136,137]. Several clinical studies were conducted in the populations of overweight or obese adults and children to examine associations between oxidative stress markers and cardiometabolic parameters, as well as the presence of CMDs or a risk of developing CMD, e.g., [131,132,133,134,135,136,137,138,139]. In general, the levels of oxidative stress markers were abnormal in obese individuals, both healthy and those with CMDs, compared to non-obese controls [138,139,140,141,142,143,144,145,146]. Moreover, measurement of various oxidative stress markers can capture different stages of oxidative stress development, which may result in various types and varying severity of tissue damage. While some markers are more specific for early stages of oxidative stress development, which are characterized by the production of ROS (e.g., H_2_O_2_), the others are related to later and more severe stages, such as ROS-mediated lipid peroxidation (e.g., IsoPs) or DNA damage (e.g., 8-hydroxy-2′-deoxyguanosine) [97,109,111,139,140,141,142,143,145,147,148]. More advanced and intense oxidative stress may be associated with a more likely occurrence of endothelial dysfunction, atherosclerotic vascular changes, or symptomatic CMDs. Importantly, the findings of some studies suggest a usefulness of oxidative stress markers for identifying individuals with increased risk of development or progression of CMDs. Specifically, IsoPs, which are markers of lipid peroxidation and have vasoconstricting and inflammatory properties, were shown to be useful for predicting HTN [139,145,147]. An increase in prevalence of childhood obesity over recent years, which may contribute to increased cardiometabolic risk in children populations, resulted in expanding clinical research addressing an occurrence of oxidative stress in children populations, especially those with obesity [1,139,140].

### 5.1. Systemic Essential Hypertension

The presence of oxidative stress was demonstrated in both animal and human models of HTN [149,150]. Associations between oxidant–antioxidant balance markers, adiposity indices, and BP values were observed in obese healthy subjects, obese individuals with elevated BP, and patients with HTN [139,140,141,142,143,144,147,150,151]. Additionally, urinary and plasma IsoPs were significantly lower in treated hypertensive men compared with the untreated men [152].

In the study of Atabek et al. [140], positive correlations between plasma concentrations of peroxy radicals and systolic BP, as well as between peroxy radicals and total cholesterol level, were found in the group of obese children, 25% of whom had hyperlipidemia and HTN. Importantly, in the control group of healthy and non-obese children, the levels of peroxy radicals were significantly lower compared to obese children. In addition, no correlations between oxidative stress markers and cardiometabolic parameters including BP values were found in the control group [140]. Furthermore, in the study of Morandi et al. [151], which included children and adolescents with obesity, total systemic antioxidant capacity (TAC) was inversely associated with systolic BP and pulse pressure, which is a marker of arterial stiffness and subclinical vascular damage. In addition, a negative association between TAC and a risk of systolic HTN was observed, which was independent of another significant predictor of HTN, i.e., BMI z-score. Importantly, in this study, the participants with HTN and the composite measure defined as “elevated systolic BP + HTN” accounted for 25% and 48% of the studied population, respectively. The relationship between oxidative stress and systolic BP could be explained by hypothesizing that oxidative stress would increase the BP by producing endothelial dysfunction, which was shown previously to be strongly correlated with systolic BP [151,153].

Several authors reported higher plasma and urine concentrations of IsoPs in obese subjects with elevated BP compared to non-obese individuals and obese subjects with normal BP [139,141,145,147,151]. Additionally, positive correlations between IsoPs and various cardiometabolic markers, such as BP, central adiposity indices (BMI derivatives and waist circumference), insulin resistance markers, body fat, total cholesterol, triglycerides (TGs), high sensitivity-C-reactive protein (hs-CRP), T2D diagnosis, and cigarette smoking were found [139,141,145,147,151].

In the study of Ostrow et al. [139], which included children with obesity, 8-IsoP correlated with mean 24 h systolic BP and was higher in subjects with “masked HTN”, defined as elevated mean 24 h systolic BP, compared to normotensive subjects. Importantly, the participants with “masked HTN” accounted for 16% of the study group [136]. In another study including 897 premenopausal overweight women with an average BMI of 27 kg/m^2^ and without a history of CVD, the urine levels of F2-IsoP and F2-IsoP metabolite (15-F2t-IsoP-M) were positively correlated with diastolic BP, central adiposity indices, T2D diagnosis, and cigarette smoking [147]. Moreover, the findings of this study suggest that elevated F2-IsoP metabolite may be considered as a predictor of an incident HTN in the long-term follow-up with a maximum of 11.5 years. Additionally, F2-IsoP was shown to be associated with diastolic BP in African American, but not in White American healthy obese youth, although positive correlations between oxidative stress and body fat were found in obese healthy young subjects of both races [145]. These findings suggest that oxidative stress may be a mechanistic link between key risk factors and occurrence of CMDs and indicate a usefulness of oxidative stress markers, such as markers of lipid peroxidation (e.g., IsoPs), for predicting the development of CVD, such as HTN [139,145,147].

However, some authors did not observe positive correlations between BP values, such as daytime mean BP, night-time mean BP, and systolic BP, and oxidative stress markers, such as IsoPs levels and NO production/metabolism markers [141,154]. Specifically, in the study of a large cohort of obese children characterized by a relatively higher composite prevalence of HTN, consisting of sustained HTN and “masked” HTN [155] (i.e., 17% in obese children vs. ~6% in the group of children with normal weight), no correlations between BP values and oxidative stress markers were observed [141]. However, in this study, oxidative stress markers were correlated with measures of obesity and insulin resistance, independently of BMI [141]. The authors concluded that elevated IsoPs levels may represent an early marker of cardiometabolic dysfunction, even in the absence of established HTN [141]. Several IsoPs actions related to vascular dysfunction, such as vasoconstriction, induction of platelet aggregation, and enhancement of adhesion of neutrophils and monocytes to endothelial cells may be involved in the pathogenesis of cardiometabolic dysfunction. Additionally, the positive associations between IsoPs and cardiometabolic risk factors, such as insulin resistance indices, inflammation markers, and atherogenic lipids were found. However, no differences in IsoPs levels between groups of overweight treated hypertensive patients, overweight untreated hypertensive patients, and normotensive controls were reported [152].

Moreover, Baráth et al. [143] observed significantly higher concentrations of MDA, another marker of lipid peroxidation, in obese patients with HTN compared to non-obese hypertensive patients, obese normotensive subjects, and healthy controls. Additionally, Minuz et al. [156] demonstrated enhanced oxidative stress and persistent platelet activation in patients with HTN and advanced vascular lesions in the course of severe hypertensive retinopathy. These findings indicate that oxidative stress markers might be useful for identifying those hypertensive patients who are at an increased risk of cardiovascular events and who might benefit from a long-term antiplatelet therapy [156]. Furthermore, the associations between the oxidative stress to DNA and BP measures were also investigated [142,148]. In the study of Yavuzer et al. [148], an increased urinary level of the 8-oxo-7,8-dihydro-2′-deoxyguanosine (8-oxodG), the oxidative stress marker of DNA damage, was found in elderly individuals with HTN compared to controls. However, no differences in the 8-oxodG between the groups of obese men with and without HTN were found in the study of Cejvanovic et al. [142]. Nevertheless, statistically significant associations between 8-oxodG and mean 24 h systolic and diastolic BP were observed.

### 5.2. Atherosclerotic Risk Factors, Atherosclerosis, and Metabolic Syndrome

Atherosclerosis is a chronic vascular disease characterized by the formation of an atherosclerotic plaque in the vessel wall of medium- or large-sized arteries, which results in the development of atherosclerotic CVD. While the pathogenesis of atherosclerosis is not well understood, extensive research indicates that atherosclerosis is a consequence of various inflammatory, oxidative, and mechanical processes. The atherosclerotic process is initiated by damage of endothelial cells, which can be triggered by oxidative stress associated with atherosclerotic risk factors, such as obesity (especially abdominal obesity), insulin resistance, elevated glucose levels in prediabetes and T2D, HTN, dyslipidemia, and cigarette smoking, all of which often coexist [157,158,159,160]. The MetS reflects the clustering of individual cardiometabolic risk factors related to abdominal obesity, insulin resistance, dyslipidemia, and elevated BP [5,10]. Accumulation of visceral fat leading to abdominal obesity results in disturbances in the production of inflammatory and anti-inflammatory cytokines, followed by the development of chronic low-grade inflammation [146,161]. Central obesity plays a significant role in the pathogenesis of atherosclerotic CVD. The oxidative theory of atherosclerosis assumes that the process of atherogenesis is significantly influenced by the oxidative modification of lipoproteins, which leads to lipid peroxidation followed by formation of foam cells and damage of endothelial cells of the vessel wall [160]. Additionally, IsoPs exert several actions that may be involved in the pathogenesis of vascular dysfunction. The IsoPs are potent vasoconstrictors in most vascular beds, induce platelet aggregation, and enhance the adhesion of neutrophils and monocytes to endothelial cells, all of which may contribute to atherosclerosis [141].

Enhanced systemic oxidative stress can be a significant mechanism linking obesity, especially central obesity, to atherosclerotic CMDs, and may contribute directly or indirectly to the development of atherosclerosis [136,146,157]. Oxidative stress may also interact with inflammatory processes in the early stages of atherosclerosis [146,161]. Positive correlations were found in healthy young subjects between oxidative stress markers (such as MDA and CD), inflammatory markers (such as CRP), measures of abdominal obesity (except for BMI), and the presence of MetS [161]. Furthermore, oxidative stress in those with obesity is involved in various interactions with metabolism and actions of the atherogenic lipids. Those interactions include a postprandial increase in TGs concentrations, oxidative modification of low-density lipoproteins (LDL) resulting in the formation of oxidized LDL (oxLDL), which has significant atherogenic properties, and a decrease in circulating high-density lipoproteins (HDL), which also have a protective effect against atherosclerosis [157,162]. These interactions may lead to further production of ROS in the endothelium, promotion of proinflammatory vascular processes, increase in endothelial damage, and development of endothelial dysfunction, which can initiate atherosclerotic processes [157]. It was shown that peroxy radical levels were higher in subjects with hyperlipidemia compared to those with normal lipid levels, while both groups had similar BMI [140]. Significant unfavorable correlations between lipid atherosclerotic risk factors were observed in overweight females with hypothyroidism across different BMI ranges [163]. These correlations include non-high-density lipoprotein cholesterol (non-HDL-C) level and the ratios of TGs/HDL-C, total cholesterol/ high-density lipoprotein cholesterol (HDL-C) and low-density lipoprotein cholesterol (LDL-C)/HDL-C, and oxidative stress markers, such as thiobarbituric acid reactive substances (TBARS) and protein carbonyls, as well as markers of antioxidant defense, such as glutathione (GSH), CAT, and GPx [163]. In addition, elevated levels of oxLDL are involved in the formation of foam cells, both development and destabilization of atherosclerotic plaque, have an association with cytotoxic and procoagulant activity, and increase expression of adhesion molecules in endothelial cells. These elevated levels of oxLDL were observed in patients with advanced atherosclerosis, such as patients with CAD and ischemic stroke [133,164,165]. Additionally, in obese men compared to slim and physically active ones, the concentration of paraoxonase-1 (PON-1) that inhibits formation of oxLDL was lower, while the levels of MDA, the lectin-like receptor of oxidized LDL type I (LOX-1), and pro-inflammatory cytokine tumor necrosis factor α were higher [146]. Additionally, in the study of patients with morbid obesity treated with bariatric surgery, the levels of oxLDL and MDA were higher in subjects with carotid atheromatous plaques detected by ultrasound examination compared to patients with no visible carotid atheroma [155]. After bariatric surgery and lowering body mass, oxLDL levels decreased in both groups but were still higher in patients with atheromatous plaques, while the levels of PON-1 and CAT were higher in patients without atheromatous plaques. Importantly, MDA levels decreased significantly in both groups of patients after the bariatric surgery, which indicated that bariatric surgery reduced lipid peroxidation independently of the presence of atherosclerotic lesions.

Impairment of endothelial function is an early disorder involved in the pathogenesis of atherosclerosis [166]. A characteristic feature of endothelial dysfunction is lowered bioaccessibility of endothelial-dependent blood vessel dilating substances, in particular NO [167,168]. An increase in total AT and abdominal fat is connected with an impairment of endothelial function dependent on NO, development of oxidative stress, and increased production of vasoconstrictor proteins [169]. Vascular NADPH oxidases are multi-subunit enzymatic complexes occurring in myocytes of blood vessels and endothelial cells, which are a major source of superoxide anion radical within the walls of blood vessels [133]. NADPH oxidases play a key role in the pathogenesis of vascular diseases including cerebrovascular accident [170]. The findings of Silver et al. [169] demonstrated that vascular endothelial cell protein expression of NADPH oxidase-p47(phox), CAT, nitrotyrosine, and phosphorylation of eNOS were greater in the overweight/obese patients than in the normal-weight patients. This may provide novel insight into the molecular mechanisms linking obesity to oxidative stress and increased risk of atherosclerotic CVD. In the study by López-Domènech et al. [171], after losing weight, a decrease in BP, improvements in metabolic parameters, reduction in inflammatory response and oxidative stress parameters in leukocytes, such as decrease in superoxide and protein carbonyl, and enhancement of antioxidant systems activity were observed, even in subjects with morbid obesity undergoing dietary intervention with calorie restriction. In addition, a significant reduction in subclinical markers of atherosclerosis, such as small and dense LDL particles, myeloperoxidase (MPO), sP-selectin, and leukocyte adhesion, was reported. These findings suggest that the improvement of oxidative stress and inflammation may be underlying mechanisms responsible for reducing the risk of CVD in obese subjects after losing weight resulting from a calorie restriction diet.

### 5.3. Coronary Artery Disease

Elevated levels of free fatty acids and triacylglycerols in obese persons favor ectopic lipid accumulation, especially in the heart [172]. The amount of epicardial AT is strongly correlated with visceral obesity. During CAD development, epicardial and perivascular AT accumulates in the myocardium around medium and small coronary arteries. This results in compression, local delivery of free fatty acids and cardioactive hormones, release of pro-inflammatory adipokines, apoptosis, coronary calcification, and atheromatous plaque formation [172]. Obese hearts are less metabolically flexible and cardiometabolic changes in the heart favor ROS generation [173]. Increased fatty acid uptake and fatty acid oxidation, mitochondria dysfunction, augmented NOX activity, and decreased antioxidant capacity enhance oxidative stress in the heart and cardiomyocytes [174]. It seems that mitochondrial dysfunction plays a key role in MetS and enhances progression of metabolic disorders [175]. Little data are available to provide evidence on elevated level of oxidative stress in the heart tissue of CAD patients. In the study of obese males aged <55 years undergoing coronary artery bypass graft surgery (CABG), Niemann et al. [175] found increased levels of oxidative stress markers, such as 8-OHdG and protein carbonyls, in cardiomyocytes, as well as disorders of mitochondrial function. These abnormalities were comparable to changes observed in older patients. Importantly, young obese patients demonstrated signs of disturbed mitochondrial function and biogenesis otherwise seen only in old patients (obese or normal-weight). In addition, the length of telomeres was significantly reduced in cardiomyocytes of obese CAD patients compared to slim ones in the same age group [175]. Additionally, mean telomere length was reduced up to 30% in cardiomyocytes of old (normal-weight and obese) and young obese subjects, with the shortest telomeres in the old obese patients. Shortening the length of telomeres constitutes a sensitive indicator of increased oxidative stress in post-mitotic cells, such as cardiomyocytes [174]. In the group of obese patients undergoing CABG, Gramlich et al. [176] found increased levels of ROS and an enhanced expression of ROS-producing enzymes (i.e., p47phox, XO), decreased antioxidant defense mechanisms, and elevated inflammatory markers (such as vascular cell adhesion molecule-1) in the right atrial myocardial tissue and in serum, which were more pronounced with increasing BMI. These findings may indicate an increased risk of developing cardiomyopathy and cardiac dysfunction in obese patients undergoing CABG due to ongoing ischemia and reperfusion related to CABG.

### 5.4. Obesity and Oxidative Stress: Direct Link to CVD Outcomes and Mortality

The interactions between obesity and oxidative stress promote endothelial dysfunction, atherosclerotic coronary lesions, LV hypertrophy (LVH), myocardial fibrosis, cardiac remodeling, and LV diastolic and systolic dysfunction. These all contribute to the pathophysiology, symptoms, and outcomes of CVD including various arrhythmias, such as AF, myocardial infarction, and HF (particularly HF with preserved LV ejection fraction) [172,173]. Associations between oxidative stress and CVD outcomes including CVD-related mortality were examined in a few clinical studies [177,178]. In a large population-based cohort study including 9949 older adults (aged 50–75 years) from Germany, the urinary levels of oxidation end products of lipids and DNA, i.e., 8-IsoP and oxidized guanine/guanosine (OxGua), were shown to be independent predictors for CVD-related mortality and stroke incidence (8-IsoP was also a predictor of fatal stroke) in a 14-year follow-up [177]. Moreover, adding these biomarkers to the European Society of Cardiology SCORE scale improved its abilities for the prediction of CVD mortality. Furthermore, both biomarkers were associated with an incidence of myocardial infarction only in obese subjects (i.e., those with BMI ≥ 30 kg/m^2^), but not in the total population. These findings provided strong evidence of the involvement of oxidative stress in the pathophysiology and outcome of CAD, as well as indicated a usefulness of 8-IsoP and OxGua measurements for prediction of myocardial infarction in obese older subjects. Several biological mechanisms might contribute to these results, such as the significant role of oxidative stress in the initiation and progression of atherosclerosis, including atheromatous plaque rupture and the enhancement of systemic oxidative stress related to the accumulation of AT during obesity. However, in another prospective cohort study conducted by Godreau et al. [178], no association between the serum level of 8-IsoP (which is not so stable as urine level) and mortality was observed in the subjects with elevated BMI in contrast to individuals with low to normal BMI.

Table 2 displays the relationships between oxidative stress markers and cardiometabolic parameters in obese subjects.

### 5.5. Oxidative Stress and Cardiometabolic Risks: Clinical Perspectives

The management of patients with CMDs is challenging yet critical. Preventative and therapeutic approaches, which aim at mitigating oxidative stress through lifestyle and pharmacological interventions, represent promising strategies for patients with a diagnosis or high risk of CMDs [113,180,181,182,183,184,185].

The use of natural antioxidant compounds, such as vitamins, flavonoids, and polyphenols, as well various diet modifications, may improve systemic oxidative status and be helpful for the treatment and prevention of CVD and MetS [180,186]. For example, there is some evidence that the antioxidant capacity of green tea, cocoa, and extra virgin olive oil is associated with cardioprotection and a decrease in the BP, making these plant-derived nutraceuticals interesting potential tools against HTN and other types of CVD [180,187]. Additionally, small clinical studies show that increasing antioxidant capacity by vitamin E supplementation improves cholesterol levels, markers of oxidative stress, arterial compliance, increases insulin sensitivity, and decreases the systolic BP and, to a much lesser extent, diastolic BP in mildly hypertensive adults [188,189,190]. However, the results of large randomized trials do not support the use of vitamins E and C for reducing cardiovascular risk in patients at high risk for cardiovascular events, i.e., patients with CVD or T2D including patients surviving recent myocardial infarction [181,191,192]. A possible explanation may be that these vitamins are not specifically targeted to the sites of ROS generation (e.g., mitochondria) and that vitamins react more slowly with ROS than ROS can interact with NO [181]. Based on preclinical studies and small human studies, numerous other antioxidant compounds, such as resveratrol, quercetin, catechins, and several others, might exert beneficial effects for prevention and treatment of CMDs. However, large cohort randomized controlled clinical trials with adequate methodology, such as rigorous inclusion and exclusion criteria, sufficient duration of intervention, and long-term follow up, are needed to provide sufficient clinical evidence for improving cardiometabolic outcomes in subjects at high cardiometabolic risk and patients with CMDs [180,181,193,194].

Weight loss interventions, both dietary and surgical, were shown to be associated with a reduction in oxidative stress and improvements of subclinical atherosclerotic markers, suggesting that these mechanisms may contribute to the reduced risk of CVD in obese subjects after losing weight [171,179]. Structured lifestyle interventions including comprehensive multicomponent intensive cardiac rehabilitation, which can be enhanced by a plant-based diet with antioxidant capacity, are critical to improving the outcome of patients with CVD and cardiometabolic risk factors [182]. Some data indicate the beneficial effects of mitigating circadian disruption on reducing cardiometabolic risks [113,183,184,195,196]. Circadian disruption itself may be secondary to various factors, such as a prolonged daily eating period or sleep disruption that may also be associated with increased oxidative stress. While a few studies on time-restricted eating (TRE), an intervention based on modifying timing and duration of daily food intake, suggest a reduction in lipid peroxidation in obese subjects, including individuals with prediabetes; the results of ongoing studies on the effects of TRE in patients with CMDs, including MetS, are warranted [183,184,185]. Available data suggest that the benefits of lifestyle modifications, including calorie restriction diets, plant diets, or TRE, go beyond the benefits of caloric restriction and weight loss; however, feasibility and sustainability of these therapeutic interventions in both clinical studies and real-world clinical practice may be limited [197]. There is a need for further clinical research including large-scale randomized controlled trials with longer duration of TRE intervention, long-term follow-up, measurement of circadian rhythms, and additional tools for recording food intake and chrono-nutrition assessment to determine the efficacy of TRE for reducing long-term cardiometabolic risk. Additionally, future clinical trials are warranted to establish the optimal protocols of intensive cardiac rehabilitation and provide tools for sustained lifestyle changes.

Pharmacological scavenging and/or preventing the generation of ROS may both be other preventative or therapeutic options to reduce deleterious effects of oxidative stress in CMDs; however, current clinical evidence on specific antioxidant pharmacotherapies is limited [181]. Nevertheless, given that angiotensin II is a key upstream trigger of ROS formation, angiotensin-converting enzyme (ACE) inhibitors, one of the most common evidence-based pharmacotherapies for CMDs, exert their beneficial clinical cardiovascular effects in part through antioxidative mechanisms. Thus, although vitamin E therapy can be regarded as a secondary therapy that scavenges already-formed ROS, ACE inhibitors can be considered as a primary therapy that blocks ROS production at the enzymatic source [181].

The antioxidant effects of antidiabetic and antiobesity medications, such as glucagon-like peptide-1 receptor agonists (GLP-1RA) and metformin, may be useful for the management of CMDs. In addition to the glucose-lowering and weight-decreasing effects, GLP-1RA affect cellular pathways involved in redox homeostasis [198]. Several in vitro and in vivo studies proved that GLP-1RA reduce ROS and protect against oxidative stress-related cell damage induced by various stress factors, such as high glucose and fatty acids, through various mechanisms, such as activating the Nrf2 signaling pathway and enhancing the expression of antioxidant and detoxification enzymes [198,199,200]. GLP-1 protects endothelial cells from oxidant injury by reducing intracellular ROS and preventing both endothelial dysfunction and excessively stimulated autophagy, possibly by restoring HDAC6 through a GLP-1RA-ERK1/2-dependent manner [200]. The antioxidant effects of GLP-1RA may be involved in protection against atherosclerosis and diabetes complications, such as diabetic cardiomyopathy and nephropathy [198,201]. GLP-1RA also reduce glycemic variability, which has emerged as a risk factor for diabetic and cardiovascular complications, possibly through enhancing oxidative stress [201,202]. Additionally, in recent years, the utility of metformin was expanded beyond the first-line treatment for T2D due to various effects related to pleiotropic mechanisms of action, including AMPK-dependent and AMPK-independent pathways [203,204]. In addition to affecting glucose and lipid metabolism, as well as improving insulin resistance and obesity, metformin was shown to restore the cellular redox balance and affect mitochondrial function. Moreover, recent in vitro and in vivo studies showed that metformin inhibits hepatic gluconeogenesis in a substrate-selective manner via a redox-dependent mechanism of action [204]. While clinical data on antioxidant effects of GLP-1RA and metformin in CMDs are limited, further studies are needed to better understand the mechanisms of oxidative stress protection that are independent of the effects on glucose metabolism or body weight.

Despite a large amount of evidence on unfavorable effects of oxidative stress in obesity and CMDs and beneficial effects of antioxidant therapies in preclinical studies, further basic and clinical research is needed to investigate the oxidative stress-related molecular mechanisms involved in the pathophysiology of CMDs, and demonstrate established benefits of antioxidant therapies for the prevention and treatment of CMDs. There is a need to address various aspects associated with a translational gap between the preclinical and clinical phases of developing and implementing antioxidant therapies in CMDs. This includes the pathophysiological complexity of CMDs, singular molecular targets of antioxidant agents, low bioavailability of natural antioxidants, clinically irrelevant dosages of compounds in experimental studies, design of experimental studies that do not adequately reflect human populations including subjects with various comorbidities, and a lack of established knowledge on mechanisms of the switch from protective oxidative signaling to deleterious oxidative stress [180]. Antioxidant therapies represent an approach that can potentially have translational impacts leading to improvements in health and a reduction in risks for CMDs, disability, and premature death; however, more clinical evidence on benefits provided by such therapies is warranted. Additionally, the mechanisms of the beneficial effects of antioxidant therapies are still poorly understood. Extensions of study protocols adding the broader spectrum of relevant biomarkers and mitochondrial function evaluation are desirable.

## 6. Conclusions

Cardiometabolic diseases (CMDs), such as CVD, MetS, and T2D are associated with increased morbidity and mortality. The growing prevalence of CMDs is mostly attributed to the aging population and common occurrence of risk factors, such as high systolic BP, elevated plasma glucose, and increased BMI, which lead to the global epidemic of obesity, MetS, and T2D. Oxidant–antioxidant balance disorders largely contribute to the pathogenesis and outcomes of CMDs, such as HTN, atherosclerosis, CAD, cerebrovascular disease, and MetS. Enhanced and disturbed generation of ROS in excess AT during obesity may lead to increased oxidative stress. Understanding the mechanisms linking adiposity and oxidant–antioxidant balance disorders to the pathogenesis and clinical outcome of CMDs is of great importance to improve the management of patients with CMDs and guide further basic and clinical research.

Expanding the knowledge on adiposity-enhanced oxidative stress related to cardiometabolic disorders can have translational impacts leading to the identification of beneficial lifestyle interventions and the development of novel effective pharmacotherapies, which can reduce the CMDs burden. Future basic research and clinical trials are needed to further examine the molecular mechanisms of adiposity-enhanced oxidative stress in CMDs and efficacy of antioxidant therapies for reducing risk and improving the outcome of patients with CMDs.

## Figures and Tables

**Table 1:** Population- and gender-specific cut-off values of waist circumference for the diagnosis of metabolic syndrome.

Population (Country/Ethnic Group)	Waist Circumference [cm]	
	Male	Female
Europid origin, Sub-Saharan Africa, Eastern Mediterranean, Middle East	94	80
United States of America	102	88
Asia, South and Central America	90	80

**Table 2:** Relationships between selected oxidative stress markers and cardiometabolic parameters in obese subjects with cardiometabolic disorders.

Oxidative Stress Marker	Results of Clinical Studies	Refs.
Isoprostanes	Higher concentration in hypertensive than normotensive subjects Positive correlations with: mean 24 h systolic BP, systolic BP, diastolic BP (in African Americans but not in White Americans), central adiposity indices, body fat, total cholesterol, TGs, total cholesterol/HDL-C, T2D diagnosis, insulin resistance markers, hs-CRP, cigarette smoking Negative correlations with: peak oxygen consumption (VO₂ max) Predictor for: HTN in a long-term follow-up, myocardial infarction in a long-term follow-up	[139,141,146,147,151,154,177]
H_2_O_2_	Positive correlations with: central adiposity indices, interleukin-6	[139]
Peroxy radicals	Higher concentration in subjects with hyperlipidemia than without Positive correlations with: systolic BP, total cholesterol	[140]
TAC	Negative correlation with: systolic BP, pulse pressure Positive correlations with: pulse wave velocity	[141,151]
MDA	Higher concentration in obese hypertensive patients compared to obese normotensives, non-obese hypertensives, and healthy non-obese controls	[143,179]
TBARS	Positive correlations with: non-HDL-C, TGs/HDL-C, total cholesterol/HDL-C, LDL-C/HDL-C	[163]
Protein Carbonyls	Higher concentration in CAD obese patients than in patients with normal weight Positive correlations with: non-HDL-C, TGs/HDL-C, total cholesterol/HDL-C, LDL-C/HDL-C	[163,175]
RBC GPx	Negative correlations with: non-HDL-C, TGs/HDL-C, total cholesterol/HDL-C, LDL-C/HDL-C	[163]
8-OHdG	Higher concentration in CAD obese patients than in patients with normal weight Positive correlations with: mean 24 h systolic and diastolic BP Predictor for: myocardial infarction in a long-term follow-up	[142,175,177]

Abbreviations: (BP) blood pressure, (CAD) coronary artery disease, (HDL-C) high-density lipoprotein cholesterol, (hs-CRP) high-sensitivity C-reactive protein, (HTN) systemic essential hypertension, (H_2_O_2_) hydrogen peroxide, (LDL-C) low-density lipoprotein cholesterol, (MDA) malondialdehyde, (RBC GPx) glutathione peroxidase in red blood cells, (TAC) total anti-oxidant capacity, (TBARS) thiobarbituric acid reactive substances, (TGs) triglycerides, (T2D) type 2 diabetes, and (8-OHdG) 8-hydroxy-2′-deoxyguanosine.

## Data Availability

Not applicable.
